# Outcome of solid and cavitary pulmonary nodules in rheumatoid arthritis patients—case series

**DOI:** 10.55730/1300-0144.5514

**Published:** 2022-04-19

**Authors:** Aysun AKSOY, Derya KOCAKAYA, Yasemin YALÇINKAYA, Emine BOZKURTLAR, Sait KARAKURT, Emel ERYÜKSEL, Nevsun İNANÇ

**Affiliations:** 1 Department of Internal Medicine, Division of Rheumatology, Faculty of Medicine, Marmara University, İstanbul, Turkey; 2 Department of Pulmonary Medicine and Critical Care, Faculty of Medicine, Marmara University, İstanbul, Turkey; 3 Department of Basic Sciences, Division of Pathology, Faculty of Medicine, Marmara University, İstanbul, Turkey

**Keywords:** Biologic drugs, cavitary nodule, leflunomide, rheumatoid arthritis, solid nodule

## Abstract

**Background/aim:**

Rheumatoid pulmonary nodule can be detected in up to 32% of rheumatoid arthritis (RA) patients and approximately one-third of nodules may cavitate. We aimed to evaluate characteristics of patients with RA developing cavitary pulmonary nodular (CPN) lesions under disease-modifying antirheumatic drugs (DMARDs), follow-up of both cavitary and solid nodules, and their outcome with the treatment.

**Materials and methods:**

RA patients who presented with CPN lesions during follow-up were recruited retrospectively in this case series analysis. Total numbers and mean diameters of cavitary and solid nodules in each thorax computed tomography (CT) have been determined and followed up by two experienced pulmonary physicians. Moreover, changes in treatment after the development of the CPN lesions and characteristics of cavitary nodules were collected.

**Results:**

Eleven patients with CPN lesions were reported. At the time of CPN diagnosis, more patients were taking leflunomide than methotrexate (81% vs 19%). Half of the patients were receiving biologic therapy and only 18% were taking anti-TNF drugs. After a median of 24 (3–65) months of follow-up, the regression of CPN lesions was determined in 45% (5/11) of patients. Four of these 5 (80%) patients were switched to a treatment regimen without leflunomide and three of them to nonanti-TNF biologic treatment or targeted synthetic DMARDs (tocilizumab, tofacitinib, and rituximab).

**Conclusion:**

CPN lesions seen in RA patients are often pulmonary manifestations of the underlying disease; however, one must rule out malignancies or infections. If lesions progress under DMARDs, it is advised to discontinue synthetic DMARDs (LEF/MTX) and switch to another biological DMARD with different modes of action

## 1. Introduction

Rheumatoid arthritis (RA) is a chronic inflammatory disease typically involving small joints. Pulmonary involvement is the most common extraarticular manifestation of the disease. Pulmonary disease, which is a major source of morbidity and mortality in RA, manifests most commonly as interstitial lung disease (ILD), airways disease, rheumatoid nodules (RN), and pleural effusions [1].

The prevalence of rheumatoid pulmonary nodules, also called necrobiotic nodules, ranges from less than 0.4% in radiological studies to 32% in lung biopsies of patients with RA [2]. These nodules can sometimes cavitate.

Nodules usually present a diagnostic challenge rather than therapeutic. Nodules in RA patients should be evaluated similarly to those in any other patient presenting with solitary or multiple pulmonary nodules, as nodules may reflect the presence of infection, malignancy, or other inflammatory diseases. Biologic drugs are now widely used for treating RA. Various side effects, mainly infection, have been described with these drugs. The efficacy of these agents for the treatment of pulmonary involvement of disease has not been specifically evaluated in large placebo-controlled randomized trials.

We report characteristics of 11 patients with RA developing cavitary pulmonary nodular (CPN) lesions under disease-modifying antirheumatic drugs (DMARDs) and follow-up of cavitary nodules and their outcome with the treatment were reported.

## 2. Materials and methods

RA patients who presented with CPN lesions during follow-up between September 2009 and April 2019 in rheumatology outpatient clinic were recruited retrospectively in this case series analysis. Diagnosis of the CPN lesions was made with computed tomography (CT) scan and indication for imaging of these patients were constitutional/respiratory symptoms or any abnormality at routine annual conventional radiography during biological treatment. Characteristics of the patients, positron emission tomography (PET), and biopsy (if available) findings were collected from patient reports. Patients without biopsy were the ones who did not accept biopsy or who had contraindication for biopsy (with cardiac failure and arrhythmia treated by the cardiac pacemaker). In addition to these, changes in treatment after development of the CPN lesion and characteristic of cavitary nodules were collected. Outcome assessment focused mainly on the number and characteristics of all nodules (solid, cavitated) with the modification of treatments, but we also evaluated cavitary nodules separately. Total numbers and mean diameters of cavitary and solid nodules in each CT have been determined with the agreed decision of two experienced pulmonary physicians, who were aware of the clinical data of the patients. An increase in total nodule count or new cavitation of a solid nodule at CT was accepted as a progression of the lesion. It is also accepted as a progression of the lesion if there is a 20% or more increase of mean nodule size even though total and cavitary nodule count is stable. A decrease in total or cavitary nodule count and a 30% or more decrease in mean nodule size is accepted as regression of the lesion.

The study was approved by the local clinical research ethics committee (reference number: 09.2019.592)

## 3. Results

### 3.1. Patient demographics

Table 1 summarizes demographic and clinical characteristics of patients. Eleven patients (5 males and 6 females) were reported with a median age of 51 (30–61) years at diagnosis and median disease duration of 17.5 (8–30) years. Eight patients had a history of smoking, four patients had subcutaneous rheumatoid nodules, and five had erosive deforming joint involvement. Nine patients (81%) were under the treatment of a median of 82 (10–100) months with leflunomide (LEF) and 2 (19%) patients had 90 (80–100) months methotrexate (MTX) usage when CPN lesion was detected. Seven out of nine patients with LEF treatment had a history of methotrexate (MTX) treatment; two patients had a history of rapid skin nodulosis with MTX. Cumulative MTX dosage was less than 1 g for 3 patients. Concomitant with conventional disease-modifying antirheumatic drugs (csDMARDs), 6 (54%) patients received biologic treatment. One patient was receiving rituximab (median duration: 12 months), 3 patients were receiving abatacept (median duration:10 (7–18) months), and 2 patients were receiving tumor necrosis factor (TNF α) inhibitors (infliximab, etanercept) (median duration: 69.5 (48–91) months).

Before initiation of biologic drugs (except rituximab), each patient was screened for tuberculosis and underwent conventional radiography, clinical pulmonary examination, and ppd/QuantiFERON test, and tuberculosis prophylaxis (9 months of isoniazid) was given to 5 of them as the results of these tests.

Two patients received antituberculosis treatment after the detection of CPN; the negative QuantiFERON test turned positive during abatacept treatment in 1 patient (case 4) and biopsy of the nodule was necrotizing granuloma for the other one although tuberculosis bacilli were not detected (case 8). After antituberculosis treatment, there was no regression at the cavitary nodules.

### 3.2. Clinical symptoms and laboratory findings

Respiratory symptoms were reported by 2 patients and constitutional symptoms by 2 patients while the others were asymptomatic during the diagnosis of CPN. All patients were seropositive, 2 patients had p ANCA (antinuclear cytoplasmic antibody)-positive, but the ELISA test for anti-MPO (myeloperoxidase) and PR3 (proteinase) were negative. Extensive examinations for microbial organisms (mycobacteria and opportunistic infections) were all negative (including cultures from bronchoscopy and bronchoalveolar lavage).

### 3.3. Imaging findings

A total of 48 CTs were evaluated by two experienced pulmonary physicians. We are presenting CT findings showing cavitary and solid nodules of our patients in Figure. The median numbers of total cavitary and solid nodular lesions were 8 (3–41) and 12 (1–61) at the beginning and after follow-up, respectively. Moreover, numbers of cavitary lesions were 1 (1–4) and 2 (0–7) at the beginning and after 24 (3–65) months of follow-up, respectively.

Fluorodeoxyglucose (FDG) PET/CT scan examination of nodules was performed to exclude malign conditions in 7 patients. We determined that both mediastinal lymphadenopathy (LAP) and cavitary-solitary nodules had increased uptake with a standardized uptake value (SUV) of 1.6–7.9 FDG. Salivary gland adenocarcinoma was detected in one of the patients with 12 FDG uptake value at the submandibular area, but biopsy from mediastinal lymph node (11 FDG) showed tuberculosis-negative necrotizing granuloma.

### 3.4. Patient outcomes

We evaluated CTs of patients for the total (solid/cavitary) nodules at the end of 28 (3–65) months follow-up and 4 (36.3%) patients were diagnosed with regressed lesions while 7 (63.7%%) patients had progressive lesions compared to previous CTs (as shown in Table 2). At the time of CPN diagnosis, more patients were taking LEF than MTX as csDMARD (81% vs 19%). Half of the patients were receiving biologic therapy and only 18% (2/11) were receiving anti-TNF drugs. After a median of 24 (3–65) months of follow-up, the regression of CPN lesions was determined in 45% (5/11) of the patients. Four out of these 5 (80%) patients were receiving a treatment regimen without LEF when their last CT was performed. Three of them were taking nonanti-TNF biologic treatment or targeted synthetic DMARDs (tocilizumab, tofacitinib, and rituximab), and one of them was receiving azathioprine (as shown in Table 2).

## 4. Discussion

We are presenting the largest case series so far in the literature with 11 seropositive RA patients with long disease duration who were diagnosed with CPN in their follow-up. Moreover, our study has an advantage of presenting the long-term follow-up of cavitary lesions in RA patients, whereas the literature generally focuses on short-term consequences of treatments. Our patient profile is compatible with the literature where rheumatoid nodules/cavitary lesions are more common in patients with smoking, long disease duration, and seropositivity.[3] After the exclusion of malignancy, vasculitis, and infection, rheumatoid nodules had been followed with CT, but treatment was mainly based on joint activity. Patients with CPN diagnosis were mostly taking leflunomide and biologic treatment. At 2 years follow-up time, 45% of CPN cases recovered. Almost all the improved cases were not using synthetic DMARDs (LEF/MTX) and taking non-TNF biologic treatment or targeted synthetic DMARDs (tocilizumab, tofacitinib, and rituximab).

The rheumatoid nodule is one of the characteristic lesions of RA and is regarded as a systemic feature of the disease. Histologically, it is a granuloma and activated macrophages are prominent within the rheumatoid nodule. These macrophages in the lesion are the likely source of the proinflammatory cytokines interleukin-1beta and tumor necrosis factor-alpha [4]. This would be consistent with evidence that immunologically mediated granulomas in other diseases. These granulomas are dynamic structures that rely on persisting effective T-cell immunity and recruitment of monocyte/macrophages to maintain the granuloma [5]. Rheumatoid nodules may cavitate in approximately one-third of cases, presumably due to ongoing vasculitis with ischemic necrosis [6]. We know that drugs used to treat the joint activity of RA can result in lung disease [7]. Progression and acceleration of rheumatoid nodule formation are well-known following MTX and LEF therapy in RA patients. The most accepted mechanism of MTX in RA patients is on the adenosine pathway promotion, which increases the activation of all types of adenosine receptors. Adenosine has a beneficial effect on reducing inflammation by the A2A receptor and by the A1 receptor it caused the promotion of multinucleated giant cell formation by human monocytes, which was suggested to be the cause of MTX induced nodulosis in RA patients [8]. LEF suppress the inflammation in synovium of RA patients by inhibiting the mitochondrial enzyme dihydroorotate dehydrogenase of activated T-cells [9]. However, the exact mechanism, by which LEF causes pulmonary nodules/CPN is unclear. Most of our patients were under LEF treatment during the detection of CPN. Like our results, previous case reports published in the literature detected CPN during LEF treatment [6, 10–12]. Among our patients, 3 out of 9 discontinued LEF and recovered after they received tocilizumab, tofacitinib, and azathioprine treatment. Two of 6 patients who continued to take leflunomide recovered, but the rest 4 were progressed. Two of the progressed cases on leflunomide received abatacept as a biologic treatment. Reducing T-cell immunity or its interaction with other critical factors such as TNF α may cause loss of structure in the rheumatoid nodule [13]. Perhaps, we may think that induction of immunosuppressive drugs that affect T-cell inhibition, such as abatacept and LEF, may lead to disruption of granuloma results in a cavitation of nodules.

The positive effects of rituximab treatment on pulmonary rheumatoid nodules were reported by Glace et al. [14]. One of our patients with CPN lesions received rituximab and all cavitary nodules disappeared at 6 months. Here, rituximab was administered to all patients, and nodules disappeared in eight patients, or the sizes were reduced significantly. MTX or LEF therapy was not discontinued after starting rituximab treatment. In contrast with the study by Grace et al., 80% of our patients with regressed CPN lesions were not receiving LEF or MTX at their last visit.

Reports show that the effect of TNF α on inflammatory mechanisms in rheumatoid nodules is less effective compared to joints, so treatment with TNF α inhibitors cannot treat pulmonary nodules [15]. In our study, 2 patients were diagnosed with CPN during anti-TNF therapy although their joint activities were under control. After discontinuation of these drugs, the number of cavitary nodules decreased, but the number of solid ones increased. A patient with CPN switched to certolizumab pegol therapy after abatacept treatment, and though joint activity improved, new cavitary nodules progressed. It is still controversial whether this is a result of the ineffectiveness of anti-TNF therapy on nodules or disease/nodule natural progression [5]. Toussirot et al. reported a case series for 11 RA patients who had pulmonary solid and cavitary nodules under the treatment of anti-TNF. After they discontinued the anti-TNF therapy, stability or even resolution of the nodular lesions had been determined, but 2 of these 3 patients with complete resolution received rituximab treatment. They have seen the development of nodular lung lesions in a patient with the reintroduction of anti-TNF therapy. [13] Although Toussirot et al. described that none of the solitary nodules progressed even though anti-TNF therapy was continued in 4 patients, we determined an increase in size and number of cavitary and solid nodules in a patient followed for 2 years under the treatment of anti-TNF therapy and leflunomide. Derot et al. [16] reported definitive regression of rheumatoid nodules after the suspension of etanercept therapy. Glace et al. [14] reported ten cases with pulmonary rheumatoid nodules that developed during the administration of traditional DMARDs and/or anti-TNF therapy.

There is a case report by Andres et al. [17] where pulmonary nodules were successfully treated by tocilizumab. With a CPN lesion, one of our patients showed regression in these lesions with tocilizumab treatment. Some case reports have suggested a response to high-dose steroids for pulmonary nodules [18]. Among our patients none of the patients with the outcome of stable/regressed lesions received high-dose steroids.

There are case series of RA patients presenting with rheumatoid nodules in the literature, but data on CPN lesions is very limited. Alpay-Kanıtez et al. presented two RA patients with CPN and a literature review of 11 cases. At the time of CPN detection, 8 and 4 patients were under LEF and MTX treatment respectively [10]. In line with these 11 cases, most of our patients with CPN lesions were using leflunomide. With the discontinuation of LEF/MTX, regression of CPN lesions was observed in most of the 11 cases in this literature review. Similarly, almost all the patients in our case series were not using synthetic DMARDs (LEF/MTX). In contrast to these previous cases with CPN lesions, none of our patients were treated with corticosteroids and/or cyclophosphamide treatment. In addition, we determined recovery of the lesions in our patients who were taking non-TNF biologic treatment or targeted synthetic DMARDs (tocilizumab, tofacitinib, and rituximab).

Eight patients underwent PET/CT scan to rule out malignancy and nodules showed mild-moderate FDG uptake. There are reports in the literature that determine a modest increase in FDG uptake of RA nodules and our results support them [19]. Therefore, PET/CT cannot distinguish inflammation from malignancy. However, 1 patient was diagnosed with submandibular adenocarcinoma under PET/CT guidance but biopsy from mediastinal lymph node (11 FDG) showed tuberculosis negative necrotizing granuloma. Therefore, the PET/CT scan can be useful to choose which nodule is more efficient for biopsy or to find out if there is another organ pathology.

Our study has some limitations. We did not have the disease activity of all the patients during the CT scan because of the retrospective design of the study. In a previous study, no relationship was reported between nodules and disease activity [11]. We also presented the initial CT scans and treatments of patients with cavirtary lesions, but we do not know exactly when the cavitary lesions occurred. The evaluators of the CT findings were not blinded to clinical data and interclass correlation was not calculated for them. However, they reached total agreement for the progression and regression of the disease. We did not have histopathology from all nodules, but we followed up the patients for a median of 24 (3–65) months and none of the lesions progressed to other diagnoses. Additionally, core needle biopsies should be interpreted carefully because the histology of the rheumatoid nodule has marked overlap with granulomatous infection and Wegener granulomatosis [20].

In conclusion, CPN seen in RA patients are often pulmonary manifestations of the underlying disease; however, one must not rule out malignancies or infections. If these lesions develop under DMARDs especially with LEF and biologic treatments with anti-TNF therapy, it is advised to discontinue synthetic DMARDs (LEF/MTX) and switch to biological DMARDs with different modes of action. In addition, improvement of the CPN lesions might be seen with non-TNF biologic treatment or targeted synthetic DMARDs (tocilizumab, tofacitinib, and rituximab).

## Figures and Tables

**Figure:**
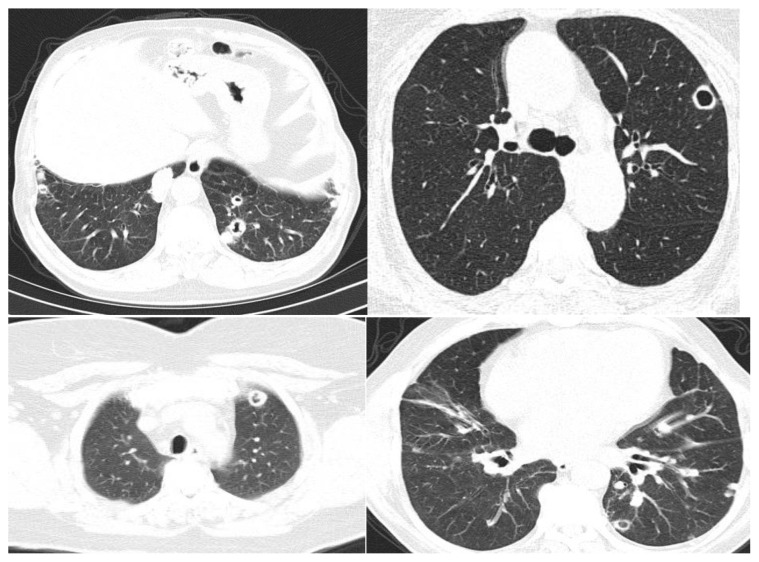
Solid and cavitary nodule of our patients.

**Table 1:** Demographic and Clinical Characteristic of Patients

Case	Sex,Age	Disease duration *Months*	Follow-up duration-*months*	Smoking-pack/*years*	Clinical characteristics	RF (IU/mL)	Anti CCP (IU/mL)	Clinical symptoms	PET-FDG (SUV)	Biopsy
Case1	F, 43	276	100	20	SN, EDJI	169	>200	Cough	-	
Case 2	M, 68	100	67	50	EDJI	NA	330	-	FDG (−)	
Case 3	F, 53	140	68	-	SN, EDJI	629	163	-	Mediastinal LAP-FDG (4.1), cavitary nodules-FDG (3.5)	Lung tru cut biopsy: Necrosis, fibrosis, and chronic inflammation
Case 4	M, 57	240	38	-	-	44	111	-	Cavitary nodules FDG (1.9) mediastinal LAP FDG (3.4)	
Case 5	F, 71	132	100	-	-	36	14	-	Mediastinal LAP-FDG (3.9), cavitary nodules (3.8–7.6)	Lung tru-cut biopsy: Chronic inflammation riched in plasma and lymphocytes, fibrosis
Case 6	M, 67	145	120	10	SN	269	21	Weight loss		
Case 7	M, 63	264	48	90	-	85	64	Weight loss	Nodules: (1.9–2.9)	
Case 8	F, 62	360	80	40	SN	282	539	Cough	Mediastinal LAP (11), maxillary bone (12.6)	Lymph node excisional biopsy: Necrotic granuloma formation containing hyaline fibrosis
Case 9	M, 71	NA	0	25	-	34	114	-	NA	
Case 10	F, 62	180	180	35	EDJI	82	>200	-	NA	Nodule excisional biopsy: necrotic granulomas containing micro abcess
Case 11	M, 65	120	120	10	EDJI	620	36	-	Nodule (3.3), mediastinal LAP (4.6)	

NA: Not available, M: Male, F: Female, SN: Subcutaneous Nodule, EDJI: Erosive Deforming Joint Involvement, LAP: Lymphadenopathy, PET: Positron Emission Tomography, RF: Rheumatoid Arthritis, anti CCP: Anti Citrullinated Peptide

**Table 2:** Characteristics of Nodules, Disease Activity, and Prognosis with Different Treatment Agents

	CT	Number of total nodules	Number of cavitary nodules	Mean size of nodules (mm)	CRP/Sedimentation (mm/hour)	DAS28/CRP	Outcome of nodule	Treatment during the CT scan
**Case1**	baseline	3	2	10.66	5/29	NA		ABT, LEF
	6 months	4	2	7.75	3/18	NA	Progression	steroid, CERT, LEF
	24 months	5	5	11	9.6/NA	NA	Progression	CERT, LEF
	30 months	7	3	11.85	7/37	NA	Progression	No treatment
	34 months	7	6	9.85	5/37	NA	Progression	LEF
	45 months	3	0	6.33	5.5/NA	3.84	Regression	Tocilizumab
**Case2**	baseline	8	1	4.5	3/7	0.96		RTX, LEF
	3 months	8	0	4.5	0.7/9	0.96	Regression	RTX, LEF
**Case3**	Baseline	5	4	18.4	17/60	2.59		ETN, LEF
	3 months	5	3	19.8	64/71	4.17	Regression	LEF
	18 months	1	0	6	4.8/NA	1.6	Regression	Tofacitinib
**Case 4**	baseline	12	1	4.75	20/38	NA		ABT, LEF
	10 months	12	2	5.25	16/31	2.11	Progression	LEF
**Case 5**	baseline	5	4	17.4	22/52	NA		LEF, ABT
	5 months	5	5	19.6	92/94	NA	Progression	LEF; ABT
	9 months	10	5	15.2	158/86	NA	Progression	LEF; ABT
	21 months	15	6	14.4	63/68	2.97	Progression	LEF; ABT
	28 months	15	7	14.2	86/71	NA	Progression	LEF, ABT
**Case 6**	baseline	10	1	3.2	3.2/37	1.5		MTX, IFX
	11 months	14	1	4.92	27/89	3.97	Progression	LEF (11th month)
	19 months	32	2	7.4	18/43	2.08	Progression	LEF, ABT (3rd months)
	33 months	29	5	6.27	11/24	2	Progression	LEF
**Case 7**	baseline	13	1	4.31	NA/NA	NA		LEF
	17 months	14	2	5.28	79/31	NA	Progression	ABT, LEF
	24 months	15	3	5.23	6/38	2.15	Progression	LEF
**Case 8**	baseline	43	1	4.91	6.7/60	NA		LEF
	12 months	61	4	7.18	4/70	NA	Progression	LEF, ABT
**Case 9**	baseline	5	2	10	4/51	1.54		LEF; LDS
	12 months	5	3	10.6	5/24	1.61	Progression	LEF,SZP
**Case 10**	baseline	3	1	2.66	5.7/28	1.65		LEF
	11 months	3	0	2.66	NA	NA	Regression	AZA
	21 months	3	0	2.66	2.9/33	1.41	Stable	AZA
	36 months	4	0	2.75	NA	NA	Progression	AZA
	51 months	4	0	2.75	14/66	1.96	Stable	AZA
	65 months	4	0	2.75	0.16/32	0.96	Stable	AZA
**Case 11**	Baseline	14	1	8.14	14/42	1.93		MTX, LDS
	3 months	14	2	8.85	14/47	NA	Progression	MTX, LDS
	18 months	17	2	7.76	30/76	3.66	Progression	LEF (6 months), LDS
	33 month)	17	3	8.09	21/51	1.66	Progression	LEF, RTX, LDS
	48 months	15	0	8.66	6/50	1.66	Regression	RTX (started 20 months ago)

AZA: Azathioprine, ETN: Etanercept, LDS: Low Dose Steroid, LEF: Leflunomide, MTX: Methotrexate, ABT: Abatacept, CERT: Certolizumab, CT: Computed Tomography, IFX: Infliximab, RTX: Rituximab
